# Impact of the Presenting Symptom on Time Intervals and Diagnostic Routes of Patients with Symptomatic Oral Cancer

**DOI:** 10.3390/cancers13205163

**Published:** 2021-10-14

**Authors:** Pablo Ignacio Varela-Centelles, Daniel Pérez López, José Luis López-Cedrún, Álvaro García-Rozado, Pablo Castelo Baz, Amparo Romero-Méndez, Juan Seoane

**Affiliations:** 1 Department of Surgery and Medical-Surgical Specialities, Medical & Dental School, University of Santiago de Compostela, Entrerríos s/n, 15705 Santiago de Compostela, Spain; pabloignacio.varela@usc.es (P.I.V.-C.); daniel.perez.lopez@rai.usc.es (D.P.L.); pablo.castelo.baz@usc.es (P.C.B.); mariaamparo.romero@usc.es (A.R.-M.); 2 Service of Oral and Maxillofacial Surgery, A Coruña University Hospital (CHUAC), Xubias-84, 15006 A Coruña, Spain; jose.lopez.cedrun@sergas.es (J.L.L.-C.); Alvaro.Garcia-Rozado.Gonzalez@sergas.es (Á.G.-R.)

**Keywords:** early diagnosis, time intervals, diagnostic delay, primary care interval, symptomatic oral cancer

## Abstract

**Simple Summary:**

Information about the relative length of patient stays, primary care, and prereferral intervals (from symptom onset to specialist referral) is very scarce, and how the presenting symptoms influence these intervals and referral routes remains unknown. This study assesses the impact of presenting symptoms on time intervals, number of visits at the primary care level, and referral pattern of patients with symptomatic oral cancer. This approach will allow targets to be identified for future interventions and the optimization of the treatment pathway for symptomatic oral cancer patients.

**Abstract:**

This investigation was aimed at determining the time intervals from the presenting symptoms until the beginning of oral cancer treatment and their relative contribution to the total time, and to assess the impact of the presenting symptom on diagnostic timelines and patient referral routes. A cross-sectional, ambispective study was designed to investigate symptomatic incident cases. The Aarhus statement was used as a conceptual framework. Strategies for minimizing potential recall biases were implemented. A sample of 181 patients was recruited (power: 99.5%; α = 0.05). The patient interval reached 58.2 days (95% CI, 40.3–76.2), which accounted for 74% of the whole prereferral interval and for more than one third of the total time interval. The presenting symptom (trigger for consultation) influenced both the number of primary care consultations and the length of time to diagnosis. General dental practitioners generated longer intervals to diagnosis (*p* < 0.005) and needed more consultations before referring a patient (RR = 0.76; 95% CI, 0.61–0.93), than general medical practitioners. The current study identifies the patient as the main target for interventions to improve awareness and reinforces the need for increased alertness amongst healthcare professionals about presenting symptoms of oral cancer and to diminish the number of prereferral consultations in order to optimize the primary care interval.

## 1. Introduction

Oral and pharyngeal neoplasms combined are the seventh most frequent cancer and the ninth-leading cause of cancer deaths worldwide [1,2]. Growing incidences have been observed for these tumors in Eastern and Central Europe and the USA [2,3].

The wide variations observed in incidence (up to 20-fold) in different geographical areas, or even within the same country for minorities or subpopulations, can be explained by dissimilarities in socioeconomic status and regional differences in risk factors [3,4,5]. In addition to the 177,757 new cases reported worldwide in 2020, an increase in incident cases of over 40% is expected for the next 20 years (280,539), along with the subsequent associated mortality [1], which unveils a global public health problem.

Despite improvements in diagnostic and therapeutic procedures, about half of oral cancers have already reached an advanced state (III or IV) when diagnosed, which implies a poor five-year survival rate (20–50%), with only minor improvements (<5%) in the last 20 years [6,7]. This circumstance has been attributed to delayed diagnoses [6,7]. In this vein, it has been suggested that early diagnosis is the most important factor influencing survival to oral cancer and that early diagnoses and treatments would be linked to survival rates beyond 80% after five years for oral cancer [8]. On the other hand, long time intervals (“delays”) to the diagnosis and treatment of symptomatic oral cancers—as well as breast, melanoma, colorectal, and also head and neck cancers—seem to be associated with poorer outcomes [9,10]. In addition, several meta-analyses disclosed diagnostic delays (>1 month) as being associated with advanced disease stage at diagnosis [9,10,11].

In order to improve the quality of research on this topic, methodological protocols have been developed in the last decade to study the journeys of symptomatic cancer patients within the model of pathways to treatment (from symptom onset to the start of treatment) framework, which allows time intervals and their prognostic implications to be identified, together with allowing intervention strategies to be designed and to minimize bias [12,13,14,15]. One result of these strategies is the finding that the longer the diagnostic-to-treatment interval, the poorer the overall survival for patients with oral squamous cell carcinomas [16]. In addition, patient delay influences survival of head-and-neck carcinomas [17,18], and diagnostic delay is a risk factor for mortality from head and neck cancer [9,11]. In particular, patients experiencing referral delay have shown a strong association with poor survival [17,19]. However, the tumour growth rate acts as a confounder when studying the liaison between delayed diagnosis and survival and it may justify the inconsistencies identified when measuring this association [9,20].

Conversely, and despite the fact that the patient interval may represent the main part of the total time interval to diagnosis and treatment, available information about the relative length of this interval, as well as about the primary care interval and the prereferral interval (from symptom onset to specialist referral), is very limited [15,21,22,23,24,25].

Although symptoms can intuitively condition both patient and primary care intervals as well as referral routes, there is no information on this issue, which is crucial for early diagnosis research [26]. Therefore, the aims of this investigation were to determine the time intervals from the first symptom (presenting symptom) until the beginning of treatment of oral cancer patients and their relative importance and to assess the impact of the presenting symptom on diagnostic timelines and patient referral routes.

## 2. Materials and Methods

A cross-sectional, ambispective, hospital-based study was designed in which the prospective component began when patients contacted the treating specialist.

Participants were recruited from among the incident cases in the 2015–2019 period with pathological diagnosis of oral squamous cell carcinoma at the CHUAC and POVISA hospitals in Galicia (North-Western Spain). Both hospitals are reference centers for oral cancer treatment under a public, free and universal healthcare scheme (Galician Health Service). The inclusion criterion was symptomatic patients, those whose physical (oral) changes or symptoms prompted them to seek care from a primary care health professional. Exclusion criteria included prevalent or recurrent cases, multiple carcinomas, secondary primary tumors, metastatic cancer, patients who had been treated at some stage at private clinics, patients with records of hospital admissions from hospital accident and emergency services, patients referred because of casual findings during unrelated consultations or as a consequence of screening programs.

These criteria permitted the identification of 280 cases during the study period, and a sample of 181 patients were recruited (participation rate: 64.6%).

**Figure 1:**
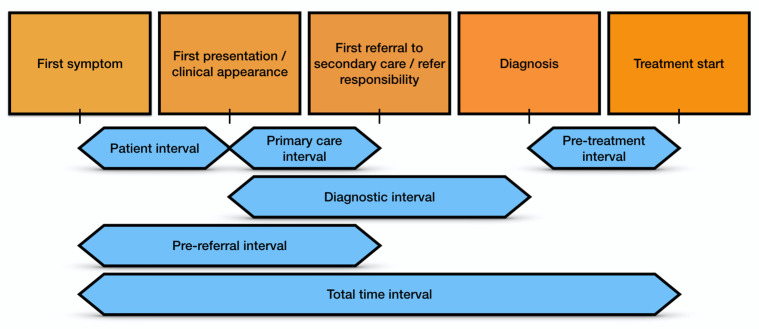
The model of pathways to treatment of symptomatic cancer patients: Aarhus Statement.

The model of pathways to treatment of symptomatic cancer patients and the Aarhus Statement were used as the conceptual framework for this investigation [12,13,14]. The intervals considered in this study were the patient interval (time from symptom onset to first consultation with a healthcare professional); the primary care interval (time from first consultation to referral for further investigation); and the overall prereferral interval (the time elapsed from symptom onset to referral and the number of prereferral consultations) [12,15,22]. The pretreatment interval (from diagnosis to start of treatment) and the overall time interval (from first symptom to the beginning of treatment) were also considered (see Figure 1) [12].

The presenting symptom was defined as the first symptom reported at presentation at a primary care setting by a patient later diagnosed with an oral squamous cell carcinoma [15]. Symptoms were recorded at the time of diagnosis by the treating specialist using a structured questionnaire. All patients in the study answered the questionnaire. In order to minimize potential memory bias, the information reported by the patient was checked against clinical records at the primary care level and also with patients’ relatives. In case of inconsistencies, this information was discussed with patients letting them know the presenting symptoms recorded in their previous clinical records until a consensus was reached. For patients referred with more than one symptom, the oral and maxillofacial surgeon asked the patient to identify the first symptom, and this information was double-checked against the individual’s primary care clinical records. For those cases with multiple symptoms, these symptoms were added together, and the resulting number was considered a variable in the study. The number of consultations was quantified by disclosing the number of consultations related to the presenting symptom using the Galician Health Service electronic medical records (Ianus^TM^) and its codification system (International Classification of Primary Care [ICPC-2 Plus]).

Finally, to compare dentists’ (GDPs) versus physicians’ (GPs) performance in the referral of oral cancer patients, the proportion of patients referred by each figure, the number of consultations at the primary care level, and the time intervals associated to each referral pattern were analyzed.

This investigation complies with the requirements of the Declaration of Helsinki and was approved by the Galician Research Ethics Committee (Ref. 2014/604).

### Statistical Analysis

The estimated value for *f*^2^ according to the adjusted model, with a sample size of 181, is 0.988. The power for detecting significant effects in at least one of the variables is 99.5% with an alpha of 0.05.

For normal continuous variables, mean and standard deviation and/or 95% confidence intervals were estimated, while the median and interquartile range were obtained for noncontinuous variables. Raw frequencies and percentages were estimated for categorical variables. Differences in interval means regarding the referral pattern were tested with the Mann–Whitney–Wilcoxon test.

The odds ratios (ORs) and their 95% confidence intervals were estimated to analyze the association between presenting symptoms and referral pattern from primary care. The relationship between the number of prereferral consultations with presenting symptoms and referrals was evaluated through risk ratios at a 95% confidence interval. For variables with few events, ORs were estimated using small-sample adjustment: the most effective modification to the estimator of OR in small sample was the following: OS(ss) = ad/(b + 1) (c + 1).

Likelihood ratio tests (LRTs) for comparing models with and without confounders (null model) were estimated. Null hypothesis for the test is that the simplest model (model without confounder) is better, so a *p*-value greater than 0.05 favors the simplest model. Models without possible confounders were always selected by LRT (Supplementary File S1).

Generalized additive models (GAMs) were used to assess the effect of selected symptoms, number of prereferral consultations, number of symptoms, and type of primary care referral pattern. GAMs are extensions of generalized linear models that allow flexible effects of continuous covariates over the response using splines. Cubic splines were used for modelling the effects of number of consultations and symptoms. The response variable was log-transformed. All statistical analysis were performed with R statistical software [27].

## 3. Results

The study included a sample of 181 patients (63.9% males; mean age 65.8 ± 12.7 years-old), with carcinomas located on the tongue (C02: 45.2%) and less frequently on the palate (C05: 9.6%), floor of the mouth (C04: 8.3%), gums (C03: 7.6%), base of the tongue (C01: 3.1%), and other sites within the oral cavity (C06: 26.1%).

The majority of patients were diagnosed at advanced disease stages (TNM III-IV: 56.7%), and total time interval from initial symptom to start of treatment was $\bar{x}$ = 159.8 days (95% CI, 136.6–182.9). The prereferral interval (time until the patient was sent for hospital care) was the largest contributor to the time spent on the patients’ pathway to treatment ($\bar{x}$ = 96.0 days; 95% CI, 70.8–121.1). In addition, the patient interval (signs/symptoms detection till consultation with a primary healthcare professional) averaged 58.2 days (95% CI, 40.3–76.2), which accounted for 74% of the prereferral interval and for more than one third of the total time interval in the Aarhus framework (Table 1).

Together, physicians (GPs) and dentists (GDPs), took mean of 28 days (95% CI, 14.3–41.81) and two consultations (IQR: 2–3) to refer patients for specialized care, making the primary care interval the shortest interval in the study.

The presenting symptom (trigger for consultation) influenced both the number of consultations at the primary care level and the time until diagnosis (Table 2 and Table 3). Pain (27.6%), ulceration (24.8%), and lumps (22.1%) were the most frequent presenting symptoms. Presenting symptoms different from white patch (14.9%) showed frequencies below 3.5%. While pain was linked to longer primary care intervals and more consultations (RR: 1.39; *p* < 0.001), lumps (RR: 0.75; *p* < 0.001) were associated with shorter diagnostic intervals and fewer consultations. The number of consultations at the primary care level showed significant positive correlation with the primary care interval (0.54 [0.36–0.68]; *p* < 0.001) and with the diagnostic interval (0.38 [0.20–0.54]; *p* < 0.001) (Table 2).

The healthcare professional who first received the patient conditioned both the diagnostic interval and the number of consultations at the primary care level. Physicians (GPs) needed a significantly lower number (OR: 0.66; 95% CI, 0.47–0.92) of consultations (Median: 2; IQR: 2.0–3.0) than GDPs (dentists) (Median: 3; IQR: 2.72–4.0), resulting in a shorter primary care interval (*p* = 0.029) (Table 3 and Table 4).

However, GDPs generated more efficient in-hospital routes by referring these patients to oral and maxillofacial surgery services, resulting in significantly shorter total time intervals (*p* = 0.05) (Table 5).

Preliminary analysis did not show modifications of the effect of the presenting symptoms or number of consultations over primary care interval when possible confounders were included in the model (age, gender, TNM-Stage, co-morbidity). Variables that did not show significant effects were not modified. Anova tables for fixed and flexible effects of the models including confounders are detailed in Supplementary File S1. 

**Figure 2:**
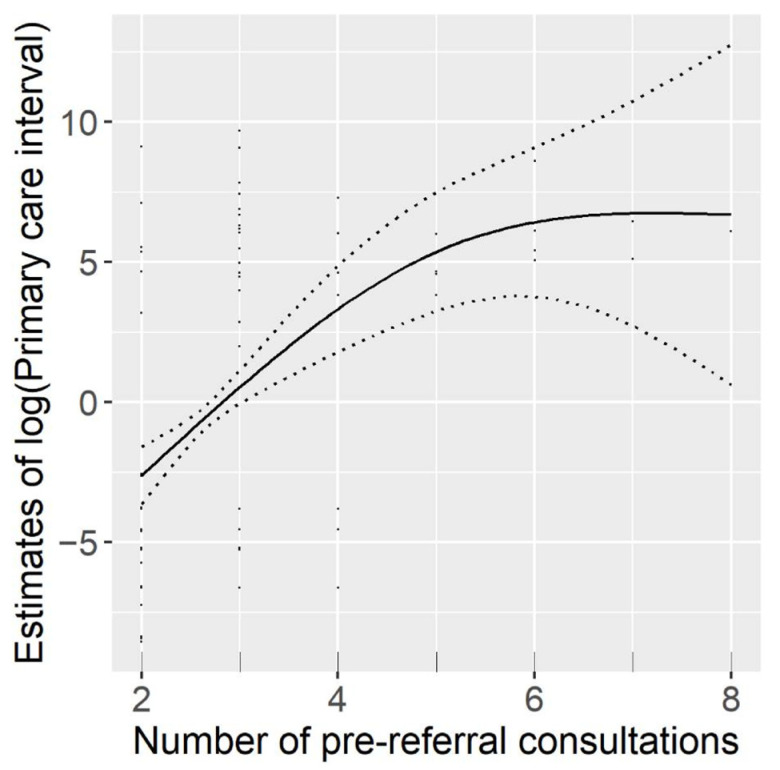
Flexible effect of number of prereferral interval on logarithm of primary care interval (mean effect centered at 0). Dotted lines representing 95% credible interval.

In the multivariate model for the primary care interval, gender and age were included in the initial model, but no effects on this interval were observed. Moreover, the LRT favored the model that excluded gender and age (LRT = 1.81; *p* = 0.403). Multivariate regression analysis identified the presenting symptom “lump” as being associated with a shorter primary care interval. Conversely, an increase in the number of prereferral consultations was linked to longer primary care intervals (Figure 2 and Table 6).

## 4. Discussion

The current study reports comprehensive quantitative data on the impact of the presenting symptom on each time interval in the pathways to treatment of symptomatic oral squamous cell carcinoma patients and on their referral routes. Our study provides relevant information for both clinicians and policymakers. The patient interval accounts for most of the prereferral and primary care intervals, and the most frequent presenting symptoms influence the number of consultations at the primary care level and thus the primary care interval. The referring units also condition the intervals and patients’ routes to treatment.

### 4.1. Strengths and Limitations

The main strengths of our study are the use of a conceptual framework for improving the design and reporting of studies on early cancer diagnosis (Aarhus Statement) [12], the designation of clearly defined events and time intervals and the use of an ambispective design, which increased the quality of the data collected. In addition, detailing information about the relative contribution of each interval to the overall time interval will allow for prioritization of interventions aimed at diminishing delays.

As these kind of studies gathers information about all time intervals in patients’ journeys from the detection of a bodily change, fully prospective designs are virtually impossible. Potential recall biases were prevented by double-checking the information provided by patients against details given by their relatives and the data recorded in primary care clinical charts. Comorbidity may cause both misattribution and a poor recording of the presenting symptom, although this phenomenon was not observed in our sample. Conversely, our sample may be affected by selection bias because it is hospital-based (participation rate: 64.6%), but this bias is highly unlikely because the features of the sample are very similar to those of the incident cases who declined the invitation to enter the study and to those of the general population with oral cancer [1]. In addition, and despite the fact that an early diagnosis and treatment of symptomatic cancer depends on many individual and health system-related factors, there is no evidence about differences in the relative frequency of the presenting symptoms of oral cancer across different countries. Our findings may be particularly relevant for regions with universal health coverage schemes with primary care gatekeepers. Patients were recruited before the onset of the COVID-19 pandemic, avoiding the impact of this new core contributing factor which conditions the self-management and help-seeking attitudes of patients and affects both referrals and appointments and shapes the planning and scheduling of treatment. Although data are scarce, several short communications have reported fewer oral cancer diagnoses during the pandemic, as well as a lack of control of potentially malignant oral disorders and an increase in the proportion of cancers diagnosed at advanced stages and longer therapeutic delays compared to the same period of the previous year [28].

### 4.2. Time Intervals and the Relative Length of “Patient Delay”

In order to improve both study design and comparability among studies on early cancer diagnosis, previous researchers in the field have recommended the use of the Aarhus guidelines [12]. Some reports that have applied this conceptual framework and used heterogeneous criteria suggested that “patient delay” is the most important contributor to delays in the diagnosis of oral cancer [25]. Reports from the Netherlands and Finland have described patient delays shorter than 1.5 months [17,19,29], while others undertaken in the UK, USA, Australia, India, and Iran have reported durations exceeding three months for this interval [25,30,31]. However, these studies show marked inconsistencies, even within the same country [19,32], probably due to the utilization of heterogeneous criteria and to the absence of a conceptual framework. In addition, symptom recognition—crucial in the patient interval—depends on the cultural and social characteristics of the patient, which hinders comparisons between populations [13,33].

The current study reports an average patient interval (80 days) that is shorter than the average reported by a quantitative systematic review [25], but its relative length compared to the primary care interval is markedly longer, which casts light on an issue for future interventions, as this also occurs with other neoplasms (breast, melanoma, testicular, vulval, cervix, or endometrial) [15]. The patient interval accounts for more than a third of the total time interval.

Little research has been conducted to investigate the primary care interval, and developed countries display the shortest intervals (<1 month) [25,34], as shown by our results, whereas the longest delays are reported from countries with weaker healthcare systems [35], although, wide, above-average intervals (187 days) have been identified in highly developed countries (Australia, USA) [25,30,36]. In addition, oral cancer treatment requires complex planning during the pretreatment interval. Surprisingly, this interval is not usually considered in studies about early diagnosis and treatment [37,38].

### 4.3. Presenting Symptoms and Time Intervals

Reports on the impact of symptoms on diagnostic timeliness have been restricted to a handful of carcinomas (breast, colon, lung, and pancreas) [26], and there is no information available about oral cancers. However, recognition of symptoms seems to be a particularly relevant factor for this neoplasm and paramount for the patient interval [13].

Oral ulcerations are one of the most frequent presenting symptoms of oral cancer (31–51%) [20,33] and were present in about one quarter (24.8%) of the patients in our study. It is worth mentioning that there are no pathognomonic signs or symptoms of oral cancer, and nonhealing ulcers, sores, or changes in symptoms may prompt patients to seek help [13,39]. The same applies to other early signs, which frequently include plain, changes in color and texture and/or precursor lesions (leukoplakia, erythroplakia) [39,40] (18.2% in our series). Misinterpretations of these bodily changes usually result in longer appraisal intervals, with a paramount influence in the total time to diagnosis [40,41].

### 4.4. Prereferral Interval (GP vs. GDP)

Oral cancer is the only neoplasm which can be referred for specialized care by both GDPs and primary care physician GPs [31]. Both types of healthcare professionals refer patients in similar proportions (GPs: 50% [13–86%]; GDPs: 40% [15–80%]) [19]. This referral pattern shows wide regional differences, even within the same country. The highest proportions of dentists referring for specialized care were reported in the UK [41], Australia [42], Denmark [43], the USA [44], Japan [45], and Argentina [37], without showing significant differences in terms of delay when compared to GPs [19]. However, our results indicate significant differences in the primary care interval and the prereferral interval depending on the referral pattern: patients referred by GDPs had shorter prereferral intervals. In addition, most dentists referred directly to hospital oral and maxillofacial surgery, ensuring a more efficient pathway through the healthcare system with shorter time intervals.

The reasons patients chose a GP or a GDP for consultation when experiencing possible symptoms of oral cancer is not known, although financial reasons or tumour sites may have played a role [46]. A recent community-based study showed that patients with a persistent oral ulceration—the most frequent presenting symptom—preferred consultation with a GP [47]. In addition, the number of consultations before referral has been proposed as a subrogated indicator of the primary care interval [48], especially for “harder to suspect” cancers (multiple myeloma, or pancreatic or stomach carcinomas), which generate more consultations and longer primary care intervals [22,48]. However, the association between the number of prereferral consultations and the primary care interval for oral cancer revealed by our results may make this indicator useful for “intermediate or easier to suspect” neoplasms.

On average, patients had two or three consultations before referral [23], and our results show GPs needed a significantly lower number of consultations and had shorter primary care intervals than did GDPs, probably because the latter undertook further investigation or “treatment trials” (removing irritating factors such as ill-fitted dentures, etc.) [23,36,49]. In addition, patients with oral ulcerations were mainly seen by GPs and seem to have experienced fewer consultations. Other less well-known patient-mediated factors and, though unlikely a factor, suboptimal clinical training of GDPs should also be considered as potential causes for the higher number of consultations by GDPs. In Spain, GPs and GDPs refer patients for hospital services independently. In contrast, referral within the primary care system may cause delayed referrals for hospital treatment.

### 4.5. Practical Implications for Research, Clinical Practice, and Health Policy

An overview of the existing research on the patient interval reveals a high risk for bias, which can be minimized by using an adequate theoretical framework, and the “Model of Pathways to Treatment” (Aarhus Statement) is highly recommended [12,13,14]. In this vein, the processes of symptom interpretation need further research and the use of validated questionnaires for collecting data from patients is strongly recommended [40].

Increasing patient awareness may have an important impact on the patient interval, and priority should be given to interventions focused on increasing public knowledge of cancer symptoms and risk factors [9] to decrease the burden of oral carcinomas. These interventions have proved more effective if based on theoretical models [50] and addressed to high-risk groups in a way that incorporate the sociocultural environment of the community.

These interventions should pay special attention to detailing the most frequent signs and symptoms of oral cancer (lumps or swelling and white or red patches) and to highlighting those with higher positive predictive value (e.g., nonhealing ulcerations) [36,37], as the main trigger for consultation is symptomatology [42]. The risk of misinterpreting symptoms as banal conditions should also be explained [41], and patients should be warned about the prognostic importance of seeking help quickly. In addition, reducing the number or prereferral consultations may be a useful early diagnosis initiative to reduce the primary care interval. However, a referral policy that is too broad may increase patients’ anxiety and hospital costs. Nevertheless, fast tracks have been useful in diminishing the time between referral and the beginning of cancer treatment [23]. Refining referral guidelines are necessary to clarify the roles of GDPs and GPs in the patient referral pathway, as is the implementation of new interventions aimed at reducing the prereferral interval of patients with oral cancer [27,51,52,53,54,55].

## 5. Conclusions

The patient interval is almost three times longer than the primary care interval and constitutes the major component of the prereferral interval. It accounts for about one third of the total time interval to treatment. This protagonist role has permitted its identification as a potential target for intervention to increase early diagnosis of oral cancer. The presenting symptom can influence the number of consultations needed by the healthcare professional and the length of the different time intervals to diagnosis. Moreover, time intervals are also conditioned by the referral pattern: while GDPs generate longer primary care intervals and a higher number of consultations, GPs use less efficient in-hospital routes causing longer total intervals. Therefore, a better understanding of the presenting symptoms, a reduction in the number of consultations, and the optimization of referral pathways with specific fast tracks tailored to the different healthcare environments would contribute to diminishing the time intervals until the start of treatment.

## Figures and Tables

**Table 1:** Time intervals (days) in the journey of oral cancer patients from symptom to treatment.

Variable	Mean (CI)	Standard Error	1st Quartile	Median	3rd Quartile
Total interval	159.80 (136.68, 182.95)	139.34	75.25	109.00	189.75
Patient interval	58.29 (40.38, 76.20)	96.10	7.00	31.00	61.00
Primary care interval	28.08 (14.36, 41.81)	63.25	59.00	31.00	115.00
Overall prereferral interval	96.00 (70.81, 121.19)	114.65	26.50	58.00	133.50
Diagnostic interval	70.12 (52.45, 87.80)	91.33	15.00	35.00	82.00
Treatment interval	32.25 (25.51, 38.99)	39.15	16.75	23.00	33.25
Mean ratio of the patient interval over other time intervals					
Patient interval/primary care interval	2.94 (2.57–3.31)	0.22	0.04	1.98	7.98
Patient interval/Prereferral interval	0.74 (0.64,0.83)	0.40	0.56	0.98	1.00
Patient interval/Total interval	0.36 (0.31,0.42)	0.29	0.10	0.33	0.53

**Table 2:** Time intervals and number of consultations at primary care by presenting symptom.

Presenting Symptom	Patient Interval	Primary Care Interval	Prereferral Interval	Diagnostic Interval	Number of Consultations		
					Median	RR (95% CI)	*p*-Value
Pain							
No (*n* = 131)	62.94 (108.34)	15.60 (38.11) *	99.80 (128.10)	61.25 (74.74)	2.51	1.39 (1.17–1.66)	*p* < 0.001 *
Yes (*n* = 50)	50.72 (72.44)	42.49 (81.59)	91.38 (97.37)	83.43 (111.39)	3.51		
Oral lump							
No (*n* = 141)	51.24 (71.26)	37.28 (72.25) *	95.27 (90.22)	83.70 (100.24) *	3.27	0.75 (0.63–0.89)	*p* < 0.001 *
Yes (*n* = 40)	71.67 (131.09)	8.67 (30.81)	97.48 (155.05)	39.16 (56.77)	2.46		
Oral ulceration							
No (*n* = 136)	61.66 (113.03)	30.24 (59.91)	102.96 (130.11)	80.89 (99.56)	3.20	0.84 (0.69–1.02)	*p* = 0.08
Yes (*n* = 45)	52.60 (57.98)	24.20 (69.74)	83.93 (81.89)	47.65 (67.02)	2.71		
White patch							
No (*n* = 154)	57.39 (95.22)	23.94 (55.88)	90.55 (113.66)	60.85 (82.09)	3.04	1.02 (0.83–1.26)	*p* = 0.82
Yes (*n* = 27)	62.50 (102.52)	40.52 (81.87)	116.82 (119.55)	107.24 (116.61)	2.00		
Red patch							
No (*n* = 175)	55.94 (94.15)	26.14 (62.95)	90.99 (111.68)	69.43 (92.05)	3.00	1.35 (0.87–2.09)	*p* = 0.17
Yes (*n* = 6)	100.17 (129.27)	58.80 (66.86)	173.20 (145.84)	84.00 (83.05)	3.00		
Bleeding							
No (*n* = 178)	58.21 (96.90)	28.42 (63.55)	96.54 (115.50)	71.17 (91.91)	2.98	0.65 (0.25–1.35)	*p* = 0.31
Yes (*n* = 3)	61.33 (73.53)	0.00 (NA)	74.50 (99.70)	16.50 (6.36)	3.06		
Burning sensation							
No (*n* = 178)	57.85 (96.97)	29.07 (64.20)	96.77 (116.25)	70.52 (91.99)	2.95	1 (0.72–1.38)	*p* = 1
Yes (*n* = 3)	74.33 (65.76)	1.33 (2.31)	75.67 (68.06)	56.67 (78.31)	1		
Ill-fitted dentures							
No (*n* = 178)	55.21 (90.76)	28.42 (63.55)	92.21 (110.07)	70.67 (91.96)	2.99	1.11 (0.58–2.13)	*p* = 0.74
Yes (*n* = 3)	403.00 (NA)	0.00 (NA)	403.00 (NA)	42.00 (56.57)	3.33		
Tooth mobility							
No (*n* = 179)	57.52 (96.18)	28.42 (63.55)	95.40 (115.23)	70.57 (92.03)	2.98	1.34 (0.66–2.70)	*p* = 0.40
Yes (*n* = 2)	145.00 (NA)	0.00 (NA)	145.00 (NA)	47.00 (49.50)	4.00		

(RR: Relative Risk; (*): (*p* < 0.05).

**Table 3:** Distribution of the presenting symptom and number of consultations by referral.

Presenting Symptom		Dental Referral (GDP *n* = 35)	Medical Referral (GP *n* = 66)	Odds Ratio (95% CI)	*p* Ratio	*p* Overall
Pain	No	21 (35.0%)	39 (65.0%)	Ref.		
	Yes	14 (34.1%)	27 (65.9%)	1.04.0.45;2.44]	0.934	1
Oral lump	No	27 (37.0%)	46 (63.0%)	Ref.		
	Yes	8 (28.6%)	20 (71.4%)	1.45.0.57;3.96]	0.442	0.574
Oral ulceration	No	27 (40.3%)	40 (59.7%)	Ref.		
	Yes	8 (23.5%)	26 (76.5%)	2.16.0.87;5.81]	0.099	0.146
Other symptoms:	No	26 (35.1%)	48 (64.9%)	Ref.		
	Yes	9 (33.3%)	18 (66.7%)	0.96.0.43;2.66]	0.878	1
**Number of consultations**		**Median (95% CI)**	**Median (95% CI)**	**RR (LCL-UCL)**	***p*-value**	
		3.57 (2.93–4.21)	2.71 (2.39–3.04)	0.76 (0.61–0.93)	0.008 *	

Percentages are estimated by row. GDP: General Dental Practitioner; GP: General Medical Practitioner; RR: Relative Risk; CI, Confidence interval; (*): *p* < 0.05).

**Table 4:** Description of time intervals by referral.

Variable	Dental Referral (GDP) *n* = 35	Medical Referral (GP) *n* = 66	Others Referring *n* = 80	*p*-Value
Total interval (days)	157.10 (103.92, 210.28)	166.16 (131.02, 201.30)	154.67 (115.00,194.34)	0.59
Patient interval (days)	42.45 (21.29, 63.60)	83.40 (40.08, 120.72)	34.88 (21.98, 47.78)	0.23
Primary care interval (days)	50.87 (11.33.90.41)	23.79 (8.35,39.22)	5.07 (0.67, 9.48)	0.02 *
Prereferral interval (days)	94.00 (50.97, 137.03)	109.42 (68.59, 150.25)	60.88 (31.57, 90.18)	0.05 *

(GDP: General Dental Practitioner; GP: General Medical Practitioner; Others referring: Healthcare professional outside the National Health Service Primary Care network.) (*) *p* ≤ 0.05).

**Table 5:** Influence of the pattern of referral on in-hospital routes.

Variable	Oral & Maxillofacial Surgery Services *n* = 138	Other Hospital Services *n* = 43	OR (95% CI)	*p* Ratio	*p* Overall
Dental Referral	32 (23%)	3 (6.98%)	Ref.		0.05 *
Medical Referral	47 (34.1%)	19 (44.2%)	4.10 (1.25;19.4)	0.01 *	
Others Referring	59 (42.8%)	21 (48.8%)	3.62 (1.12;16.9)	0.03	
Total interval (days)	152.2 (125.4, 179.1)	183 (136.1, 229.8)		0.05	

**Table 6:** Multivariate model for the primary care interval.

Multivariate Regression Model. Fixed Effects						
**Variable**	**Estimate**	**Std. Error**	**Conf. Low**	**Conf. High**	**Statistics**	***p*-Value**
Referral pattern: GPs	−1.408	1.367	−4.135	1.319	−1.030	0.306
Referral pattern: others	−0.004	1.858	−3.712	3.703	−0.002	0.998
Presenting symptom: pain	1.902	1.201	−0.495	4.299	1.583	0.118
Presenting symptom: oral lump	−2.785	1.277	−5.332	0.238	−2.182	0.033 *
Presenting symptom: oral ulceration	0.140	1.157	−2.169	2.449	0.121	0.904
**Multivariate regression model. Flexible effects**						
**Variable**	**Edf.**	**Ref. Edf.**	**F**	***p*-value**		
Number of symptoms	1.737	1.929	1.381	0.222		
Number of consultations	1.821	1.968	15.095	0.000 *		

(GPs: General Medical Practitioners; Edf: Effective degrees of freedom; (*): *p* < 0.05).

## Data Availability

The data presented in this study are available on request from the corresponding author. The data are not publicly available due to legal constraints.

## Supplementary Materials

The following are available online at https://www.mdpi.com/article/10.3390/cancers13205163/s1, Supplementary File S1. Anova tables of the models.
