# Supplementary material for: Impact of the Presenting Symptom on Time Intervals and Diagnostic Routes of Patients with Symptomatic Oral Cancer
Source: Cancers (Basel). 2021 Oct 14;13(20):5163. doi: 10.3390/cancers13205163 (PMC8533728; doi:10.3390/cancers13205163)
Supplement: Supplementary file 1 [file cancers-13-05163-s001.zip › cancers-1333764-supplementary.pdf]

**Supplementary File S1. Anova tables of the models**

| **Anova table for fixed effects** | | df | F-statistic | P-value |
|---|---|---|---|---|
| Model including sex | Referral pattern | 2 | 0,7234 | 0,4888 |
| | Pain | 1 | 2,6557 | 0,1078 |
| | Oral lump | 1 | 4,8343 | 0,0313 |
| | Oral ulceration | 1 | 0,0558 | 0,814 |
| | Sex | 1 | 0,305 | 0,5826 |
| Model including age | Referral pattern | 2 | 1,1891 | 0,3115 |
| | Pain | 1 | 1,5094 | 0,224 |
| | Oral lump | 1 | 3,3805 | 0,0709 |
| | Oral ulceration | 1 | 0,1625 | 0,6883 |
| | Age | 1 | 0,9503 | 0,3335 |
| Model including TNM | Referral pattern | 2 | 0,9466 | 0,3932 |
| | Pain | 1 | 3,4274 | 0,0686 |
| | Oral lump | 1 | 5,2061 | 0,0257 |
| | Oral ulceration | 1 | 0,1284 | 0,7212 |
| | TNM | 1 | 0,8148 | 0,37 |
| Model including comorbidity | Referral pattern | 2 | 0,7573 | 0,4736 |
| | Pain | 1 | 2,0043 | 0,1623 |
| | Oral lump | 1 | 5,126 | 0,0274 |
| | Oral ulceration | 1 | 0,3289 | 0,5686 |
| | Comorbidity (Charlson index) | 3 | 0,9658 | 0,4152 |
| Model including all the confounders | Referral pattern | 2 | 0,2969 | 0.7447 |
| | Pain | 1 | 0,8117 | 0.3729 |
| | Oral lump | 1 | 6,7698 | 0.0128 |
| | Oral ulceration | 1 | 0,2953 | 0.5898 |
| | Sex | 1 | 1,3812 | 0.2466 |
| | Age | 1 | 1,7869 | 0.1886 |
| | TNM | 1 | 1,9707 | 0.1679 |
| | Age | 1 | 0,2049 | 0.6532 |
| | Comorbidity (Charlson index) | 3 | 0,9183 | 0.4405 |

**Anova table for flexible effects**

| | | edf | Ref.df | F-statistic | p-value |
|---|---|---|---|---|---|
| Model including hospital | Number of symptoms | 1,8049 | 1,9617 | 2.1664 | 0.1207 |
| | Number of consultations | 1,7245 | 1,9239 | 6.7766 | 0.0067 |

| | | | | | |
|---|---|---|---|---|---|
| Model including sex | Number of symptoms | 1,7483 | 1,9363 | 1.4847 | 0.1958 |
| | Number of consultations | 1,8006 | 1,96 | 14,9759 | 0.0000 |
| Model including age | Number of symptoms | 1,7873 | 1,9546 | 1.9002 | 0.1362 |
| | Number of consultations | 1,7404 | 1,9325 | 11,1821 | 0.0004 |
| Model including TNM | Number of symptoms | 1,6908 | 1,9042 | 1.1635 | 0.2565 |
| | Number of consultations | 1,8692 | 1,9827 | 17,4915 | 0.0000 |
| Model including comorbidity | Number of symptoms | 1,466 | 1,7148 | 0.7220 | 0.3410 |
| | Number of consultations | 1,6895 | 1,9035 | 8.0395 | 0.0038 |
| Model including all the confounders | Number of symptoms | 1,5084 | 1,758 | 1,9403 | 0.1007 |
| | Number of consultations | 1,2644 | 1,4586 | 9,6874 | 0.0036 |

Likelihood ratio test (LRT) for comparing models with and without confounder (null model) were estimated. Null hypothesis of the test is that simplest model (model without confounder) is better, so a p-value greater than 0.05 favors simplest model. For possible confounders, models without them were always selected by LRT, as it is shown below:

**LRT test for comparing model with and without confounder**

| | Resid. Df | Resid. Dev | Df | Deviance | P-value |
|---|---|---|---|---|---|
| Null model | 67.761 | 1590.0 | | | |
| Model including sex | 66.756 | 1583.3 | 1.00503 | 6.768 | 0.59337 |
| Null model | 60.751 | 1447.6 | | | |
| Model including age | 59.754 | 1423.4 | 0.99742 | 24.245 | 0.30932 |
| Null model | 66.776 | 1441.3 | | | |
| Model including TNM | 65.786 | 1423.8 | 0.98954 | 17.488 | 0.36265 |
| Null model | 58.774 | 1484.6 | | | |
| Model including comorbidity | 55.919 | 1433.0 | 2.85519 | 51.599 | 0.53516 |
